# Ado-Mediated Depletion of Taurine Impairs Mitochondrial Respiratory Capacity and Alters the Chromatin Landscape of Inguinal Adipose Tissue

**DOI:** 10.3390/nu15163532

**Published:** 2023-08-11

**Authors:** Pei-Yin Tsai, Bo Shui, Seoyeon Lee, Yang Liu, Yue Qu, Chloe Cheng, Kaydine Edwards, Callie Wong, Ryan Meng-Killeen, Paul D. Soloway, Joeva J. Barrow

**Affiliations:** 1 Division of Nutritional Sciences, Cornell University, Ithaca, NY 14850, USA; pt389@cornell.edu (P.-Y.T.);; 2 Department of Biomedical Sciences, Cornell University, Ithaca, NY 14850, USA

**Keywords:** taurine, obesity, non-shivering thermogenesis, adipocytes, mitochondrial respiration, cysteamine dioxygenase

## Abstract

Non-shivering thermogenesis (NST) has strong potential to combat obesity; however, a safe molecular approach to activate this process has not yet been identified. The sulfur amino acid taurine has the ability to safely activate NST and confer protection against obesity and metabolic disease in both mice and humans, but the mechanism of this action is unknown. In this study, we discover that a suite of taurine biosynthetic enzymes, especially that of cysteamine dioxygenase (ADO), significantly increases in response to β_3_ adrenergic signaling in inguinal adipose tissue (IWAT) in order to increase intracellular concentrations of taurine. We further show that ADO is critical for thermogenic mitochondrial respiratory function as its ablation in adipocytes significantly reduces taurine levels, which leads to declines in mitochondrial oxygen consumption rates. Finally, we demonstrate via assay for transposase-accessible chromatin with sequencing (ATAC-seq) that taurine supplementation in beige adipocytes has the ability to remodel the chromatin landscape to increase the chromatin accessibility and transcription of genes, such as glucose-6-phosphate isomerase 1 (Gpi1), which are critical for NST. Taken together, our studies highlight a potential mechanism for taurine in the activation of NST that can be leveraged toward the treatment of obesity and metabolic disease.

## 1. Introduction

Obesity is defined as having a body mass index (BMI) greater than or equal to 30 kg/m^2^ and is among the current leading prevalent health issues worldwide. It is well established that obesity is linked with comorbidities such as cardiovascular disease, type 2 diabetes mellitus, and dyslipidemia [1]. According to the Centers for Disease Control and Prevention (CDC) in the United States, the prevalence of obesity increased from 30.5% to 41.9% during the period 1999–2020 [2]. Current treatment options, including calorie restriction, bariatric surgery, and pharmacotherapy, either present with poor long-term efficacy and/or serious side effects [3,4,5,6]. One attractive option to combat obesity is to take advantage of the molecular properties of thermogenic brown and beige adipocytes to raise energy expenditure by activating the non-shivering thermogenesis (NST) program. Brown and beige adipocytes contain specialized mitochondria enriched with uncoupling protein 1 (UCP1) that is able to convert the dissipation of chemical energy into heat production and maintain the core body temperature [7,8,9]. In adult humans, thermogenic brown adipose tissue (BAT) is localized to the supraclavicular, axillary, and mediastinal regions of the body. Studies have shown that activation of brown fat NST using mild environmental cold exposure periods is associated with enhanced whole-body metabolism such as increased resting metabolic rates, improved lipid and glucose profiles, and increased insulin tolerance [10,11,12,13]. While there have been significant advances in the mechanisms that govern the NST activation process such as cold environmental stimuli or the administration of pharmacological agents such as the β_3_ agonist CL 316,243, practical and safe approaches to stimulate NST in humans are still challenging [14,15]. Intriguingly, researchers have discovered that the sulfonic amino acid taurine (2-aminoethanesulfonate) has the natural ability to activate NST in rodent models to confer protection against obesity, but the mechanism is unknown [16].

Taurine is a naturally occurring amino acid derived from animal protein or by endogenous synthesis. Taurine can be synthesized from two major pathways. Synthesis can be driven by oxidizing cysteine to cysteine sulfinic acid catalyzed by the enzyme cysteine dioxygenase (CDO) which is then subsequently converted into hypotaurine via the cysteine sulfinic acid decarboxylase (CSAD) to finally generate taurine. Alternatively, taurine can be synthesized from cysteamine via an oxidation reaction catalyzed by the cysteine dioxygenase enzyme (ADO) [17,18,19,20,21]. Unlike the majority of other amino acids, taurine is not a building block of protein synthesis. Instead, it has two major reported roles in humans. The first is its role in bile acid conjugation that can complete the ionization of bile and enhance the emulsification of lipids [22]. In addition, studies have demonstrated that taurine supplementation can stimulate the 7α-hydroxylase (CYP7A1) mRNA expression to increase endogenous bile acid synthesis [23,24]. The other reported role for taurine is the modification of mitochondrial tRNAs in which a taurine methyluridine (tm^5^U) becomes incorporated at the wobble position of the anticodon loop of human mitochondrial tRNAs critical for the synthesis of mitochondrial proteins [25,26]. In addition to these defined roles for taurine, the simple sulfur amino acid has been implicated in a host of diverse physiological functions. Indeed, supplementation of taurine is positively correlated with enhanced muscle tone and strength, improved antioxidation, and protection against obesity and metabolic disease via the stimulation of non-shivering thermogenesis (NST) [16,27,28,29]. In randomized control human trials, researchers discovered a negative correlation between taurine and obesity, and they demonstrated that the supplementation of taurine can significantly decrease the body weight in obese volunteers [30,31,32,33]. Correspondingly in mouse studies, the administration of taurine to high-fat diet-induced (HFD) obese mice significantly reduced body weight gain and increased the expression of the highly metabolic thermogenic markers uncoupling protein 1 (*Ucp1*) and peroxisome proliferator-activated receptor-gamma coactivator 1 alpha (*Pgc1a*) in the inguinal and white adipocytes. Consistent with elevated standard thermogenic markers, taurine supplementation also increased oxygen consumption and body temperature, demonstrating the beneficial molecular mechanism of taurine in energy metabolism [16,34,35]. Moreover, taurine supplementation can also decrease the adipogenesis-related markers in white adipocytes, such as peroxisome proliferator-activated receptor alpha (*PPAR-1a*), peroxisome proliferator-activated receptor gamma (*PPAR-γ*), and CCAAT/enhancer binding proteins alpha (*C*/*EBPα*) [36]. The relationship between taurine and NST is reciprocal. Taurine supplementation has the ability to activate NST in mice and correspondingly, activated NST leads to the enhanced synthesis of taurine. Indeed, several studies have demonstrated that pharmacological activation of NST using the β_3_ agonist CL 316,243 can significantly elevate taurine levels in the inguinal and white adipocytes [37]. Overall, although the anti-obesity effect of taurine has been well reported, the underlying molecular mechanism of how this occurs and the association of taurine with NST are still enigmatic.

In this present study, we demonstrate that pharmacological activation of NST with CL 316,243 robustly increases the taurine biosynthetic enzymes predominately in the inguinal adipose tissue compared to other adipose depots. We further define the dependency of the cysteamine dioxygenase (Ado) biosynthetic enzyme and demonstrate that the loss of Ado significantly blunts intracellular taurine levels and impedes mitochondrial respiration in thermogenic adipocytes. Finally, we show that taurine supplementation can alter the chromatin landscape of primary inguinal adipocytes to upregulate genes linked to enhance NST and metabolic health. Collectively, our study provides novel mechanistic insight into the role of taurine in NST and positions taurine as a potential treatment option to combat obesity and metabolic disease in the future.

## 2. Materials and Methods

### 2.1. Animals

Studies on mice were performed according to the permission from the Cornell University Institutional Animal Care and Use Committee. Four-week-old wild-type male C57BL/6J mice were purchased from Jackson Laboratory (#000664) and housed at room temperature (25 °C) with 12 h cycles of darkness and light. Mice were fed ad libitum food and water. To conduct pharmacologically induced thermogenesis experiments, 5-week-old mice were acclimated to a thermoneutral environment (30 °C) for 7 days. Subsequently, mice were daily intraperitoneally injected (IP) for 7 consecutive days with either saline or 1 mg/kg of CL 316,243 (Cayman Chemical #17499, Ann Arbor, MI, USA) (*n* = 4, per treatment). Following euthanasia with carbon dioxide, brown, inguinal, and white adipose depots, along with liver tissue, were collected. Each sample was immediately flash frozen in liquid nitrogen and stored at −80 °C for further protein or RNA extractions.

### 2.2. Cell Culture

Primary inguinal adipose tissue was harvested from 3-week-old male wild-type C57BL/6J mice, followed by thoroughly chopping with scissors for 5 min and digesting with 15 mL of lysis buffer (PBS, 1.3 mM CaCl_2_, 2.4 unit/mL dispase II (Sigma-Aldrich #D4693, Burlington, MA, USA), and 1.5 unit/mL collagenase D) in a shaking water bath at 37 °C for 15 min. Lysates were filtered through a 100 μm cell strainer and spun down for 5 min at 600 g at 4 °C. After removing the digestion buffer, the stromal vascular fraction (SVF) was resuspended in adipocyte culture media (DMEM/F12 with 10% FBS, 25 mM HEPES, and 1% PenStrep) and filtered through a 40 μM cell strainer, followed by centrifuging for 5 min at 600× *g* at 4 °C. Subsequently, cells were resuspended with adipocyte culture media and plated on polystyrene cell culture plates, coated with 2% gelatin. After 48 h, cells were gently washed with PBS two times and replenished with fresh adipocyte culture media. All primary inguinal and immortalized brown adipose cell cultures were grown at 37 °C with 5% CO_2_. For adipocyte differentiation, cells were seeded on polystyrene cell culture plates with 2% gelatin coating. The following day, cells were differentiated with DMEM/F12 (supplemented with 5 μg/mL insulin, 1 μM Rosiglitazone, 1 μM Dexamethasone, 0.5 mM Isobutylmethylxanthine, and 1 nM T3). After 48 h, the medium was replaced with maintaining media (5 μg/mL Insulin and 1 μM Rosiglitazone) and replenished every two days until day 6. On the seventh day, cells were treated with 1 μM CL 316,243, PBS, or 1 mM taurine for different experimental designs.

### 2.3. Generation of Ado-KO Cells

The single guide (sgRNAs) of the CRISPR-Cas9-based Ado knockout cells were designed according to the following database https://chopchop.cbu.uib.no/ (accessed on 24 August 2021). The sgRNAs sequences are as follows: sgAdo forward: *5*′-TTCCCGGGCCGAGTACACCG-*3*′, *sgAdo reverse*: *5*′-CGGTGTACTCGGCCCGGGAA-*3*′. The sgRNAs were cloned into the LENTICRISPR v2.0 (Plasmid #52961) plasmid vector developed by Zhang’s group. The vector V2 CRISPR DNA Plasmid (1 µg) was co-transfected in 293T cells along with 3 mg of the viral envelope PMD2 (Addgene # 12259) and 4 mg of the viral packing PsPAX (Addgene #12260) plasmids using the Polyfect reagent according to the manufacturer’s instructions. The empty vector CRISPR DNA Plasmid was used as a control. After 48 h, the lentiviral supernatant was collected from the 293T cells and transduced into the immortalized brown fat cell line D.E 2.3 (gift from the Kazak laboratory, McGill University, Montreal, QC, Canada). Stable transduced cells were then established via puromycin selection (1 µg/mL).

### 2.4. Mitochondria Isolation and Seahorse

The brown fat cell line DE 2.3 cells were differentiated as described above. Cells were then scraped with digestion buffer (300 mM sucrose, 5 mM HEPES, 1 mM EDTA, pH 7.2 with KOH) and lysed with a pre-chilled glass-Teflon homogenizer (13 strokes). Cell lysates were centrifuged at 800× *g* at 4 °C for 10 min. The supernatant was then collected and centrifuged for 10 min at 8500× *g* at 4 °C to pellet the mitochondria. The supernatant was then removed, and the mitochondrial pellet was washed with 1 mL 1× MAS buffer (70 mM sucrose, 220 mM mannitol, 5 mM KH_2_PO_4_, 5 mM MgCl, 2 mM HEPES, 1 mM EGTA, pH 7.2 with KOH) and centrifuged for an additional 10 min. The mitochondria pellet was then resuspended with 100 mL MAS buffer, and the protein concentration was quantified by BCA Protein Assay Kit (ThermoFisher #23227, Waltham, MA, USA). The mitochondrial oxygen consumption rate (OCR) value was assayed by the Agilent Seahorse Bioanalyzer. For this, 10 mg of mitochondria was loaded into XFe24 cell culture plates followed by 20 min of spinning at 2000× *g* at 4 °C and ultimately refilled 450 µL MAS buffer into each well. The mitochondrial stress test compounds were then administered as follows (final concentration): Port A: Pyruvate (9 mM) and Malic acid (9 mM) (for pyruvate-driven respiration), Port B: Rotenone/Antimycin A (135 μM each). Respirometry data were collected using the Agilent Wave software v2.4. To measure extracellular acidification rate (ECAR), cells were plated and differentiated on XFe24 Seahorse cell culture plates. Cells were then washed and incubated for 30 min in unbuffered Dulbecco’s Modified Eagle’s Medium (DMEM) supplemented with 2 mM L-glutamine. We then performed the cellular glycolysis stress test by administering the following compounds: Port A: Glucose (25 mM), Port B: Oligomycin (3 µM), Port C: 2-Deoxy-D-glucose (2-DG) (50 mM). After each experiment, cells were stained with Hoechst and the cell numbers were measured using the Lionheart FX automated microscope (Agilent, Santa Clara, CA, USA).

### 2.5. Protein Extraction and Western Blot

Tissues were lysed with 2% Sodium Dodecyl Sulfate (SDS) supplemented with protease inhibitor (ThermoFisher #A32963, Waltham, MA, USA) and homogenized with metal beads for 30 min at 4 °C. Lysates were then centrifuged at maximum speed for 20 min and the protein supernatant was then retained. To extract protein from cells, adipocyte cultures were scraped with 2% SDS lysis buffer and rotated at 4 °C for an hour. The cells were then subjected to ultrasonic treatment for 10 min with 30 s on/30 s off (Biorupter, American Laboratory Trading, Groton, CT, USA) followed by 15 min of centrifugation at 4 °C at maximum speed. The protein concentrations of the both the tissue and cell lysates were assessed by BCA Protein Assay Kit (ThermoFisher #23227, Waltham, MA, USA) and protein samples were supplemented with 4× laemmli blue (Bio-Rad #1610747, Hercules, CA, USA) and heated at 37 °C for 10 min. Prepared samples were resolved on SDS-polyacrylamide gels and then transferred to PVDF membranes. A membrane was blocked with 5% milk for one hour and then washed with TBST before being incubated with primary antibodies overnight. The following day, membranes were washed with TBST and then targeted by secondary goat anti-mouse or anti-rabbit antibodies. Following the secondary staining, the membranes were washed with TBST and imaged by FluorChem system. Densitometry was conducted by applying Image J software version 1.530. The list of primary antibodies has been included in the Supplementary Materials, Table S1.

### 2.6. RNA Extraction and Real-Time Quantitative PCR

Tissues were homogenized with Qiagen TissueLyser II in Trizol reagent (Invitrogen), and cells were scraped with Trizol reagent (Invitrogen, ThermoFisher Scientific, Waltham, MA, USA). The RNA was extracted according to the manufacturer’s protocol. The concentration and quality of RNA were analyzed using Nanodrop (ThermoFisher, Waltham, MA, USA) and the reverse transcription reaction was performed by qScript cDNA Synthesis Kit (Quanta Bio). Gene expression analyses were performed by the CFX384 Real-Time PCR System using SYBR Green (Bio-Rad, Hercules, CA, USA) for the real-time polymerase chain reaction (RT-PCR). The list of primers has been included in the Supplementary Materials, Table S2.

### 2.7. Measurements of Taurine Levels

Differentiated brown fat DE 2.3 cells were washed with 2 mL PBS and processed according to the manufacturer’s protocol (Cell Biolab #MET-5071, San Diego, CA, USA) to measure the intracellular taurine concentrations.

### 2.8. Metabolomics

For untargeted metabolomics, six-week-old C57BL/6J mice were injected daily via intraperitoneal injection (IP) with either saline or 1 mg/kg CL 316,243 (Cayman #17499) for consecutive seven days (*n* = 3, per treatment). Mice were euthanized with carbon dioxide, and inguinal tissues were homogenized with 2 mL cold methanol and 4 mL of cold chloroform. Samples were then mixed with 2 mL molecular-grade water. After 5 min of incubation, samples were centrifuged at 4 °C for 10 min at 3000× *g*. The polar metabolites were transferred into new tubes and dried under nitrogen flow. Metabolites were resuspended in 30 µL 30% acetonitrile and analyzed by liquid chromatography/ mass spectrometry (LC-MS) on a Vanquish LC coupled with the ID-X MS (ThermoFisher Scientific, Waltham, MA, USA) conducted by the Harvard Center for Mass Spectrometry. Samples or standards were injected into ZIC-pHILIC PEEK-coated columns (150 mm × 2.1 mm, 5 micron particles, column temperature maintained at 40 °C, SigmaAldrich). The mobile phase was HPLC grade water, 20 mM ammonium carbonate, 0.1% ammonium hydroxide, and 97% acetonitrile. The flow rate at the first 30 s ramped from 0.05 to 0.15 mL/min and was maintained at 0.15 mL/min. All data were acquired in the ID-X polarity switching at 120,000 resolutions. MS_1_ data were acquired in polarity switching for all samples. MS_2_ and MS_3_ data were acquired by the AquirX DeepScan function for pooled samples. Results were analyzed in Compound Discoverer 3.3 (CD, ThermoFisher Scientific, Waltham, MA, USA). Identifications were based on MS_2_/MS_3_ matching with the mzVault library and corresponded to retention time built with pure standards (Level 1 identification), or on mzCloud match (Level 2 identification). For taurine-targeted metabolomics, primary inguinal cells were separately treated with 1 µM CL (Cayman #17499) or PBS for 24 h. Subsequently, cells and media were processed with the same biphasic extraction described previously. Taurine concentrations were then measured via LC-MS and analyzed the area of the exact mass for the corresponding ions of the targets as well as the intracellular labeled isotopic taurine levels (13C2, 99%; 15N, 98%, Cambridge isotope #CNLM-10253-PK).

### 2.9. Subcellular Fractionation

Differentiated primary inguinal and brown DE 2.3 adipocytes were washed with PBS and then scraped with digestion buffer (300 mM sucrose, 5 mM HEPES, 1 mM EDTA, pH 7.2 with KOH) and lysed with a pre-chilled glass–Teflon homogenizer (10 strokes). Cell lysates were centrifuged at 1200× *g* at 4 °C for 10 min. The supernatant was then collected and centrifuged for 10 min at 8500× *g* at 4 °C to separate the mitochondria. The supernatant was then transferred to a new tube and stored as cytosolic fractions. The mitochondrial pellet was washed with 500 µL mitochondria storage buffer and centrifuged at 4 °C for 10 min two times (Qproteome Mitochondria Isolation Kit #37612, Qiagen, Germantown, MD, USA).

Differentiated primary inguinal and brown DE2.3 adipocytes were washed with pre-cold PBS and scraped with 10 mL PBS. After spinning at 450 g at 4 °C for 5 min, the nuclear pellet was resuspended with lysis buffer (Qproteome Nuclear Protein Kit #37582, Qiagen, Germantown, MD, USA) and incubated on ice for 15 min. Subsequently, the sample suspension was mixed with 25 μL detergent solution NP (Qproteome Nuclear Protein Kit #37582, Qiagen, Germantown, MD, USA) and spun at 10,000 g at 4 °C for 5 min. After removing the supernatant, the pellet was resuspended with the lysis buffer, followed by centrifuging at 10,000× *g* at 4 °C for 5 min and the nuclear pellet stored at −80 °C for the protein process.

### 2.10. MitoTracker Red Staining

DE 2.3 brown adipocytes were plated in 96-well plates and differentiated as described previously. After 7 days, differentiated cells were washed with PBS three times and subsequently stained with 30 nM of MitoTracker Red CMXRos dye (Thermo Fisher Scientific #2123602, Waltham, MA, USA) for 20 min. The nuclei were then stained with Hoechst for 45 min as an indicator of total cell number. Cells were then imaged and quantified using the Lionheart FX automated microscope (Agilent, Santa Clara, CA, USA) as described previously.

### 2.11. Mitochondria Copy Number Quantification

The genomic DNA from DE 2.3 cells was isolated using the QIAamp DNA Micro Kit (Qiagen #56304, Germantown, MD, USA) following the manufacturer’s instructions. A quantitative PCR reaction was then performed using mitochondrial DNA and nuclear DNA-specific primer sequences (detailed below in Supplementary Table S2). Mitochondrial DNA copy number was then assessed as described in the study by Rooney et al. [38] using the following equation: (1) The difference of cycle threshold: $∆Ct=Ct\;\left(nuclear\;DNA\right)-Ct\;(Mitochondrial\;DNA)$. (2) The copies of mitochondrial DNA: $mtDNA=2\times 2^{∆Ct}$.

### 2.12. Nuclei Extraction and ATAC-Seq Analysis

Two million primary inguinal cells were seeded in a 10 cm culture plate and scraped with 1 mL PBS after fully differentiating. Subsequently, cells were centrifuged at 4 °C for 10 min at 200× *g* to generate cell pellets. Cell pellets were then resuspended by 200 mL homogenization buffer (60 mM Tris (pH 7.8), 30 mM CaCl_2_, 18 mM MgAc_2_, 0.1 mM PMSF, 1 mM ß-mercaptoethanol, 320 mM sucrose, 0.1 mM EDTA, 0.1% NP40) and homogenized by using plastic pestles for one minute. The samples were then mixed with 1.8 mL washing buffer (10 mM Tris-HCl (pH 7.4), 10 mM NaCl, 3 mM MgCl_2_, 0.001% Tween-20) and spun down at 4 °C for 10 min at 500 g. After removing supernatants, the cell pellets were homogenized by the plastic–Teflon in 300 µL washing buffer and filtered through 70 µm nylon filters, followed by spinning at 4 °C for 10 min at 500 g three times. The supernatant was removed, cell pellets were resuspended with transposition mix TD buffer (20 mM Tris-HCl pH 7.6, 10 mM MgCl_2_, 20% Dimethyl formamide) and filtered through 40 µm nylon filters. For nuclei quantification, the samples were treated with DAPI (4, 6-diamidino-2-phenylindole) and trypan blue in a 2:1:1 ratio, counted on a hemacytometer. The library preparation and data processing were performed in the laboratory of Dr. Paul Soloway, as described previously [39]. The nucleosomal patterning and sequencing was conducted by the Cornell Institute of Biotechnology. The Integrated Genome Viewer 2.13.1 was used to visualize ATAC-seq signal peaks.

### 2.13. Gene Ontology Analysis

Shiny GO v0.741 was used to perform the gene ontology analysis (GO) on the top 50 upregulated inguinal gene accessibilities profiles with *p*-values less than 0.05.

### 2.14. Statistical Analysis

All data were expressed as mean ± SEM, unless other specified. GraphPad Prism 9 was used for the statistical analysis to determine the difference between two independent groups by two-tailed unpaired Student’s *t*-tests. Compound Discoverer 3.2 was used to run the analysis of variance (ANOVA) and Tukey’s HSD post hoc test on the LC-MS data (Thermo Fisher). The significance level was set at *p* < 0.05. Each image legend depicts the value of n together with the statistical characteristics.

## 3. Results

### 3.1. The Taurine Biosynthetic Enzyme Ado Is Potently Upregulated in Inguinal Adipose Tissue in Response to β_3_ Adrenergic Activation of NST

**Figure 1:**
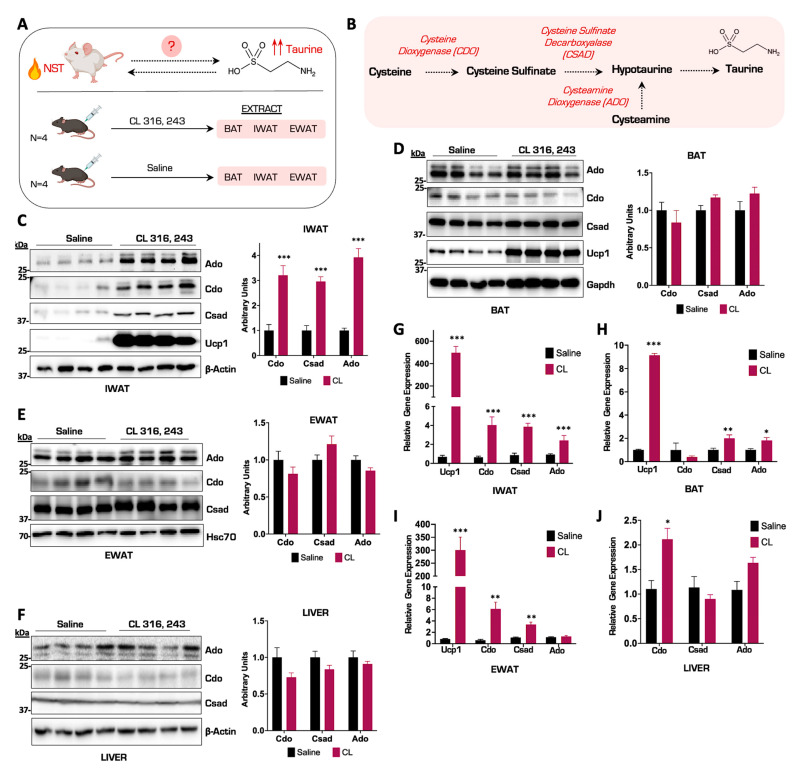
The taurine biosynthetic enzyme Ado is potently upregulated in inguinal adipose tissue in response to β_3_ adrenergic activation of NST. (**A**) Schematic of thermogenesis activation experiment in wild-type (WT) male mice (*n* = 4). (**B**) Schematic representation of the taurine biosynthetic pathway. (**C**–**F**) Representative Western blot of IWAT, BAT, EWAT, and liver tissues from mice injected with CL or saline for 7 days (*n* = 4). β-Actin, Gapdh, and Hsc70 are the protein loading controls. Densitometry of Cdo, Csad, and Ado protein levels normalized to β-Actin, Gapdh, or Hsc70 are shown on the right. (**G**–**J**) Relative mRNA expression of *Ucp1*, *Cdo*, *Csad*, and *Ado* from WT mice injected with CL or saline for 7 days in different adipocytes and liver (*n* = 4). All figures and data are represented as mean ± SEM. Significance is expressed as * *p* < 0.05, ** *p* < 0.01, *** *p* < 0.001 by Student’s *t*-test.

To investigate the molecular association between taurine biosynthesis and pharmacologically induced thermogenesis, we injected male C57BL/6J wild-type mice with either the β_3_ adrenergic receptor agonist CL 316, 243 (CL), or saline vehicle control for a period of seven consecutive days to activate thermogenesis. We then extracted brown, beige, and white adipose tissue depots in addition to liver tissue and profiled the taurine biosynthetic enzymes Cdo, Csad, and Ado to determine their response to activated thermogenesis (Figure 1A,B). From mice injected with CL, we performed a protein and gene expression profile on the three main adipose depots: brown adipose tissue (BAT), inguinal adipose tissue (IWAT), and white adipose tissue (EWAT). Immunoblot results showed that the protein thermogenic marker Ucp1 increased relative to the saline vehicle control group in BAT and IWAT in response to CL, confirming that we successfully activated thermogenesis. Protein analysis revealed a significant increase in the protein levels of the taurine biosynthetic enzymes Cdo, Csad, and Ado in the IWAT depots (Figure 1C). Interestingly, this was in contrast to the levels of the taurine biosynthetic enzymes in BAT and EWAT, which displayed no significant increases after CL stimulation compared to saline vehicle controls (Figure 1D,E). Gene expression profiling of the IWAT aligned with the protein analysis showing a potent increase in *Ucp1* and all three taurine biosynthetic enzymes *Ado*, *Cdo*, and *Csad* (Figure 1G). Curiously, the *Cdo* and *Csad* together with the *Csad* and *Ado* mRNA expression levels increased in EWAT and BAT, respectively, but did not align with protein levels (Figure 1H,I). Taurine biosynthetic enzymes are expressed ubiquitously in mammals [21] and to determine if their increase in IWAT is a specific response to NST stimulation or whether it is just a general phenomenon, we interrogated the taurine biosynthetic pathway in the liver. Immunoblot analyses indicated no changes in taurine biosynthetic enzymes in response to NST activation, indicating that the robust increases in the gene and protein levels of the taurine biosynthetic enzymes were specific to thermogenic inguinal adipocytes in response to adrenergic stimulation, arguing for a specific role of taurine in NST (Figure 1F,J). Taken together, we discovered that the gene and protein levels of the taurine biosynthetic enzymes robustly increase in inguinal adipose tissue compared to other adipose depots in response to β_3_ adrenergic activation of NST.

### 3.2. Ablation of Ado Reduces Taurine Levels and Impairs Mitochondrial Respiratory Capacity in Thermogenic Adipocytes

**Figure 2:**
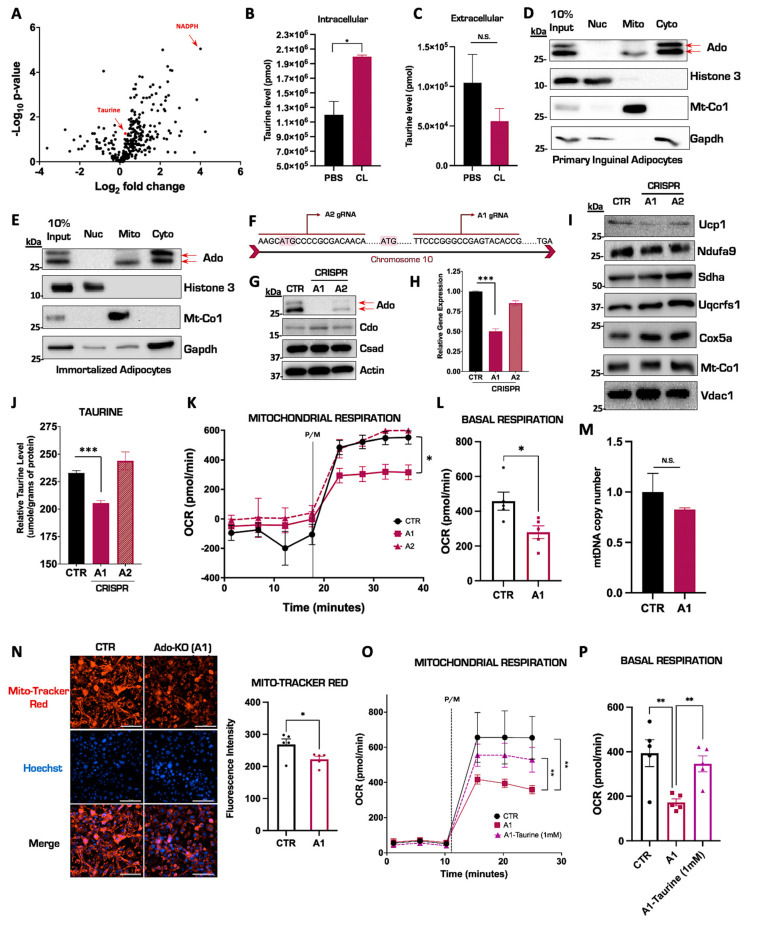
Ablation of Ado reduces taurine levels and impairs mitochondrial respiratory capacity in thermogenic adipocytes. (**A**) Volcano plot of metabolites from IWAT in mice injected with saline or CL for 7 days. The log 2-fold change was calculated based on the ratio of CL versus saline treatment. (**B**,**C**) Targeted intracellular and extracellular taurine levels in primary inguinal cells treated with CL compared to PBS controls performed by LC-MS. (**D**,**E**) Representative Western blot for Ado expression in different subcellular fractionations from primary inguinal (**D**) and immortalized thermogenic DE2.3 cells (**E**). Histone 3, Glyceraldehyde-3-phosphate dehydrogenase (Gapdh), and Mitochondrially Encoded Cytochrome C Oxidase I (Mt-Co1) represent the nuclear, cytoplasmic, and mitochondrial loading controls, respectively. (**F**) Diagram of a portion of the Ado gene sequence and sgRNA binding sites. (**G**) Representative Western blot of Ado CRISPR ablation in DE 2.3 cells. (**H**) Relative mRNA expression of *Ado* in CRISPR DE 2.3 cells. (**I**) Representative Western blot of Ucp1 and differential mitochondrial electron transport chain protein complexes from CRISPR DE 2.3 isolated mitochondria. (**J**) Intracellular taurine levels in CRISPR DE 2.3 cells. (**K**) Oxygen consumption rate (OCR) of CRISPR DE 2.3 vector control and CRISPR Ado isolated mitochondria (*n* = 5). (**L**) Quantification pyruvate and malate (P/M) induced oxygen consumption rate from CRISPR DE 2.3 control and Ado knockout isolated mitochondria. The true mitochondrial respiration was calculated by taking the basal OCR values subtracted by the antimycin/rotenone treatment (*n* = 5). (**M**) Relative mtDNA copy number in Ado KO cells compared to controls (*n* = 4). (**N**) On the left: representative fluorescent images of MitoTracker Red and Hoechst in DE 2.3 control (CTR) and CRISPR Ado KO (A1 cells). On the right: Quantification of MitoTracker Red fluorescence intensity (*n* = 5). (**O**) Oxygen consumption rate (OCR) of isolated mitochondria from CRISPR DE 2.3 control, CRISPR Ado KO, and CRISPR Ado KO cells supplemented with 1 mM taurine for 24 h (*n* = 5). (**P**) Quantification of panel (**O**). The true mitochondrial respiration was calculated by taking the pyruvate/malate (P/M) OCR basal values subtracted by the antimycin/rotenone treatment (*n* = 5). All figures and data are represented as mean ± SEM unless otherwise annotated. Significance is expressed as * *p* < 0.05, ** *p* < 0.01, *** *p* < 0.001 by Student’s *t*-test. N.S stands for non-significant.

Previous studies have shown that taurine levels can be enhanced by NST in IWAT [37], but the magnitude of this increase in relation to cellular metabolites in primary inguinal adipocytes is unknown. We therefore injected mice with CL for seven consecutive days to induce NST and performed untargeted shotgun metabolomics on isolated inguinal tissue to map taurine metabolite levels. Interestingly, compared to energy homeostasis regulators such as nicotinamide adenine dinucleotide phosphate (NADPH), the taurine metabolite levels displayed a significant, albeit modest, increase the IWAT (Figure 2A). We then postulated that perhaps the increase in intracellular taurine levels was modest because the inguinal tissue actively secretes the metabolite into circulation. To test this, we cultured and differentiated primary inguinal adipocytes and stimulated them with CL to activate NST or saline control for 24 h. We then measured taurine levels in both the inguinal adipocytes and the extracellular culture media. There, we confirmed a significant increase in intracellular taurine levels with no alterations in extracellular media (Figure 2B,C) indicating that the significant elevation of taurine in response to NST is retained in the cell for downstream biological processes. Of the three main taurine biosynthetic enzymes, Ado is the least characterized in regard to a function in brown and beige fat. We first wanted to define the subcellular localization of the Ado enzyme to gain additional mechanistic insight on taurine biosynthesis. We performed subcellular fractionation studies in primary inguinal adipocytes and isolated nuclear, cytoplasmic, and mitochondrial fractions. The Ado enzyme migrates as a doublet in immunoblot analyses yet curiously according to bioinformatic profiling, there is no recorded isoform of the enzyme. Intriguingly, subcellular fractionation experiments revealed that Ado is localized to both the mitochondrial and cytoplasmic compartments yet only the upper band of Ado localized in the cytoplasm while the lower band of Ado is present in both in the cytoplasmic and mitochondrial compartments (Figure 2D). To define the dependency of Ado on taurine synthesis, we generated CRISPR-Cas9 mediated ablations of Ado in an immortalized brown adipocyte cell line DE 2.3 [40] that displayed the same subcellular localization pattern of Ado as inguinal primary adipocytes (Figure 2E). We designed two CRISPR single guide RNAs (sgRNAs), termed A1 and A2, targeted to the first exon in Ado (Figure 2F). The Ado protein and gene levels were fully ablated in the A1 cell population while in the A2 cell population, there was a partial knockdown of Ado with no changes in the other taurine biosynthetic enzymes (Figure 2G,H). There were also no changes in Ucp1 or the mitochondrial core electron transport chain protein levels resulting from the loss of Ado (Figure 2I). To define the impact of Ado ablation on taurine synthesis, we quantified intracellular taurine levels following a 24 h CL treatment in the DE 2.3 CRISPR cells compared to vector controls. Complete ablation of Ado (A1) significantly reduced taurine levels by approximately 12% while the partial deletion of Ado (A2) did not alter taurine levels (Figure 2J), indicating that the residual levels of Ado were sufficient to maintain taurine synthesis. We next wanted to assess whether the cellular depletion of taurine levels would adversely affect mitochondrial respiratory capacity, which is a proxy for functional NST. We therefore isolated mitochondria from both Ado-KO and control brown fat cells and measured mitochondrial respiratory capacity using the Seahorse Bioanalyzer. Interestingly, compared with control and A2 cell lines, mitochondrial respiration was significantly impaired in the A1 cells, indicating that the reduced levels of taurine impaired healthy mitochondrial functions (Figure 2K,L). The ablation of Ado did not cause a decrease in overall mitochondrial content (Figure 2M), but instead, compromised mitochondrial oxidative–phosphorylation capacity as evidenced by decreased intensity of the mitochondrial membrane-sensitive MitoTracker Red staining (Figure 2N) in the Ado-KO cells compared to controls. To confirm that the decrease in mitochondrial function was due to the decline in taurine levels, we replenished taurine back to Ado-KO cells and measured mitochondrial respiratory capacity. Indeed, restoration of taurine levels significantly rescued basal mitochondrial respiratory capacity (Figure 2O,P) indicating that the Ado-mediated synthesis of taurine is important to maintain optimal mitochondrial function. Overall, we demonstrate that intracellular taurine levels significantly increase in primary inguinal adipocytes in response to pharmacological activation of NST. Furthermore, we show that Ado is localized to both the mitochondrial and cytoplasmic compartments and that CRISPR Cas9-mediated ablation of Ado reduces intracellular taurine levels and correspondingly impairs mitochondrial respiratory capacity, thus underscoring the importance of taurine in maintaining functional NST.

### 3.3. Taurine Supplementation Remodels the Chromatin Landscape in Primary Inguinal Cells

**Figure 3:**
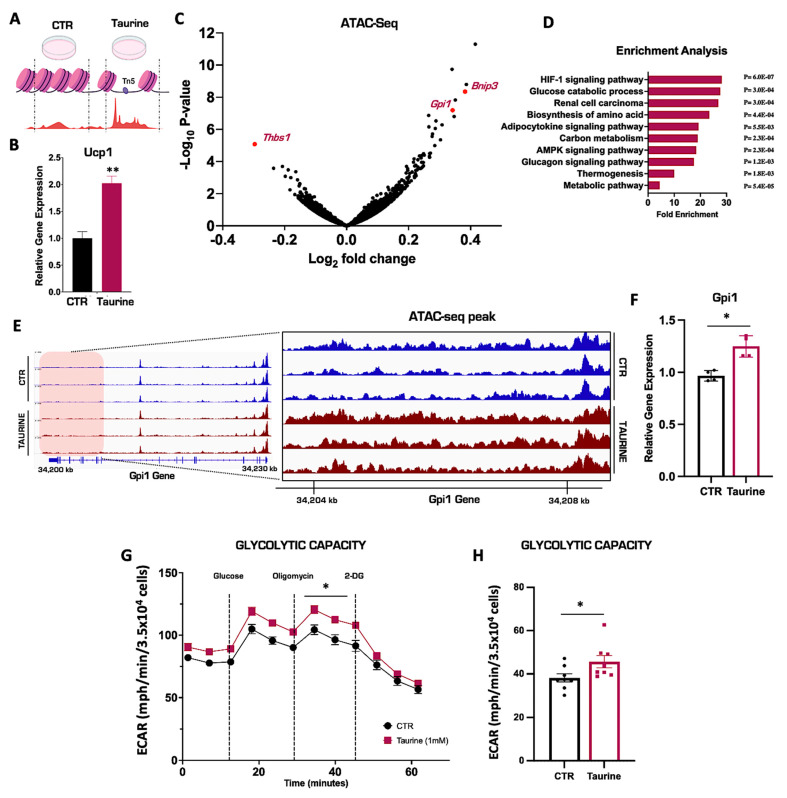
Taurine supplementation remodels the chromatin landscape in primary inguinal cells. (**A**) Schematic of ATAC-seq in primary inguinal cells treated with 1 mM taurine or PBS for 24 h. (**B**) Relative mRNA expression of *Ucp1* in primary inguinal cells treated with PBS or 1 mM taurine for 24 h (*n* = 3). (**C**) Volcano plot of differentially expressed gene accessibilities (DEGs) in primary inguinal cells. (**D**) Gene Ontology (GO) enrichment analysis of DEGs in primary inguinal cells. (**E**) Integrated Genome Viewer (IGV) images of ATAC-seq peak signal of the gene Gpi1 in primary inguinal cells treated with PBS (blue) or taurine (purple). On the left: ATAC-seq chromatin accessibility peaks in the full Gpi1 gene. On the right: zoom-in showing a specific chromatin region (shaded red region) of the Gpi1 gene to highlight differential chromatin accessibility peaks mediated by taurine treatment. Genome location of the Gpi1 gene is indicated by the kilobase (kb) markers. (**F**) Relative mRNA expression of *Gpi1* in primary inguinal cells (*n* = 4). (**G**) Extracellular acidification rates (ECAR) in primary inguinal cells treated with 1 mM taurine or PBS for 24 h. (**H**) Quantification of glycolytic capacity value. The true glycolytic capacity was calculated by taking the read of Oligomycin treatment subtracted by the 2-DG treatment (*n* = 8). All figures and data are represented as mean ± SEM. Significance is expressed as * *p* < 0.05, ** *p* < 0.01, by Student’s *t*-test.

We have demonstrated that the pharmacological activation of NST results in significant increases in intracellular taurine, but its molecular fate remains unknown. To elucidate a potential mechanistic role for taurine, we hypothesized that taurine may have the ability to alter chromatin structure similar to methionine, another sulfur-containing amino acid [41,42]. To test this, we performed an assay for transposase-accessible chromatin with sequencing (ATAC-seq) to map the genome-wide chromatin accessibility in the primary inguinal cells treated with taurine or PBS vehicle control for 24 h (Figure 3A). As reported previously, taurine supplementation significantly increased *Ucp1* mRNA levels in primary inguinal adipocytes, confirming its ability to successfully activate NST (Figure 3B) [16]. Global ATAC-seq analysis revealed that the taurine supplementation differentially remodeled the chromatin accessibility landscape in several gene loci in primary inguinal adipocytes (Figure 3C). We then performed gene ontology (GO) analyses of the genes with the most significant upregulated chromatin accessibility patterns, determined by log 2-fold change. Gene accessibility was altered in several metabolic pathways in response to taurine treatment, including accessibility changes in the glucose catabolic and thermogenic metabolic pathways (Figure 3D). One of the most upregulated differential gene accessibility patterning based on differential ATAC-seq peak patterning was the Glucose-6-phosphate isomerase 1 (Gpi1), which is an enzyme that catalyzes the reversible interconversion of fructose-6-phosphate to glucose-6-phosphate. Taurine supplementation increased the chromatin accessibility of Gpi1 (represented by higher ATAC-seq peaks heights compared to controls), which corresponded with a significant increase in Gpi1 mRNA expression in primary inguinal cells compared to PBS vehicle controls (Figure 3E,F) [43,44]. Concordant with the upregulation of glucose metabolism (Figure 3D), taurine supplementation in primary inguinal adipocytes also enhanced cellular glycolytic capacity (Figure 3G,H). This suggests that taurine supplementation can fuel mitochondrial NST by enhancing glucose metabolism. Overall, we demonstrate that taurine supplementation remodels the inguinal adipocyte chromatin landscape to increase chromatin accessibility, thereby enhancing the transcription of critical genes that promote functional NST. This may be a potential mechanism for the role of taurine in NST that can be leveraged toward the treatment of obesity and metabolic disease.

## 4. Discussion

The integrated nature of taurine and NST remain of great intrigue in the field. Taurine has the ability to activate NST and NST activation correspondingly has the ability increase the biosynthesis of taurine. Indeed, the association between taurine and the protection from obesity and metabolic disease has been well documented [16]. In the current study, we examined the taurine biosynthesis pathway in response to adrenergic activation of NST to determine which adipose depots synthesized taurine. Curiously, of the thermogenic adipose tissues profiled, the taurine biosynthetic enzymes Cdo, Csad, and Ado were only significantly increased in inguinal adipose tissue (IWAT) and not in brown or white adipose depots (Figure 1C). Inguinal adipose tissue also displayed the highest level of taurine upon NST activation. These findings align with those of the field and suggest that taurine may exert its metabolic functions in the inguinal depot.

Taurine biosynthesis can originate from two biological starting points, cysteine-driven synthesis through the Cdo enzyme or cysteamine-driven synthesis through the Ado enzyme. The enzyme Cdo has been well studied and it has been shown that the loss of Cdo in adipocytes significantly reduces taurine levels and impairs mitochondrial function by blunting mitochondrial respiration and inhibiting mitochondrial gene expression, such as cytochrome c oxidase subunit 5b (*Cox5b*), ubiquinol–cytochrome c reductase core protein 1 (*Uqcrc1*), and succinate dehydrogenase complex flavoprotein subunit a (*Sdha*) [45,46]. Our study adds to the field by providing the first evidence that the biosynthetic enzyme Ado is also critical for taurine synthesis. Ablation of Ado significantly reduces intracellular taurine levels, leading to failures in mitochondrial function through impaired respiratory capacity as mitochondrial oxygen consumption rates (Figure 2J–M). This proves that both the Cdo and Ado taurine biosynthetic pathways are critical to maintain cellular taurine levels. The question, however, still remains: what is the biological role of taurine in inguinal adipose tissue and how does this metabolite regulate mitochondrial function? Taurine has the ability to modify mitochondrial tRNAs and previous studies demonstrated that impairments in 5-taurinomethyl-uridine synthesis result in inefficient mitochondrial protein biogenesis [47]. In addition, multiple studies discovered that reduced taurine levels resulting from taurine transporter (TauT) depletion caused significant defects in mitochondria morphology, such as swelling and impaired complex I protein in the electron transport chain [48,49]. Additional studies propose that taurine may play a role in mitochondrial cristae remodeling by regulating the mitochondrial tRNA translation optimization 1 (Mto1) and the mitochondrial splicing system 1 (Ms1) proteins [50]. Indeed, it was shown that an Mto1 ablation, accompanied by diminished taurine mitochondrial RNA modification, resulted in impaired mitochondrial protein translation and a significant reduction in mitochondrial energy supply [51]. Therefore, it is possible that mitochondrial respiration is affected by the defined role of taurine in the modification of mitochondrial tRNAs and the regulation of mitochondrial cristae structure, both of which are required for the translation of mitochondrial oxidative phosphorylation (OXPHOS) proteins to generate ATP and sustain healthy mitochondrial components [52,53,54,55,56]. In our Ado loss-of-function system, however, mitochondrial dysfunction persisted despite the fact that there were no defects in mitochondrial protein translation. Indeed, there were no changes in Mt-Co1 levels in Ado knockout adipocytes compared to vector-treated controls. This would suggest that there is an alternative mechanism for taurine in the regulation of mitochondrial function. Another possibility is that loss of taurine increased mitochondrial oxidative stress and that this is what impaired mitochondrial function. A previous study observed that depletion of the taurine transporter in murine hearts decreased cellular taurine levels, which led to elevated mitochondrial oxidative stress and cellular apoptosis [52]. This will need to be explored in future studies to define the role of taurine in regulating mitochondria function.

In our studies, we noted that the taurine biosynthetic enzyme Ado migrates on immunoblot analyses as a doublet, and we confirmed using CRISPR Cas9-mediated ablation that both bands are indeed Ado (Figure 2G). It is possible that these bands represent different isoforms of Ado despite the fact that alternative isoforms are not reported for the enzyme. It is also possible that there may be post-translational modifications (PTM) on Ado such as phosphorylation or acetylation that may explain the doublet banding. Now that we have established that the Ado-derived synthesis of taurine is critical to maintain taurine metabolite levels in the inguinal depot and is essential to maintain healthy mitochondrial function, the presence of potential PTMs on Ado may shed light into the regulatory roles of this enzyme. This is particularly intriguing since the Ado bands exhibit differential subcellular localization in primary inguinal adipocytes, where the upper band is unique to the cytoplasmic fraction while the bottom band is present in both cytoplasmic and mitochondrial compartments [57,58].

It still remains enigmatic why taurine biosynthesis is elevated following either environmental or pharmacological activation of NST (Figure 2A,B) [16,37]. To our knowledge, taurine is a terminal metabolite and does not participate in any other enzymatic reaction. It also does not become incorporated into protein as the other amino acid building blocks. The function of taurine in inguinal adipocytes therefore remains elusive. In the present study, we postulated that taurine may have an impact on chromatin structure similar to other sulfur-containing metabolites such as methionine. As a DNA methylation donor, methionine can synthesize s-adenosylmethionine (SAM) and induce DNA methylation and correspondingly differential accessibility and expression in specific gene regions [59,60]. We therefore performed a genome-wide ATAC-seq analysis to determine if there are chromatin accessibility changes following taurine supplementation in inguinal adipocytes. Related chromatin state changes were observed in single cell analysis of inguinal adipose from cold- and CL-316,243-exposed mice [61]. Interestingly, we discovered that taurine supplementation can increase the chromatin accessibility of multiple genes associated with NST metabolic pathways such as glucose catabolism. As a representative example, taurine supplementation increased both the accessibility and mRNA transcription of the Gpi1 gene. Gpi1 is a catalytic enzyme involved in glycolysis and multiple studies have proved that both glucose uptake and the glycolysis pathway are enhanced under cold-induced NST in mammals [62,63]. In human studies, PET-scanning also demonstrated that the radio-labeled glucose (^18^FDG) uptake significantly increased in young healthy volunteers, while decreasing in elderly or patients diagnosed with metabolic diseases, who have been considered to have lower NST capacity [64]. It is unknown if chromatin remodeling occurs as a result of direct or indirect taurine function. One hypothesis as to how taurine could be mediating this process could be through the relationship between taurine and mitochondrial DNA. Taurine has the ability to modify mitochondrial methyluridine tRNAs to 5-taurinomethyluridine-tRNA, and this modification results in an increase in mitochondrial protein translation [51,56]. This increase in taurine-mediated mitochondrial protein translation could trigger the mitochondrial–nuclear genome crosstalk and cause a corresponding increase in nuclear gene transcription that would result in chromatin remodeling [65,66]. This hypothesis is plausible on a physiological level because an increase in mitochondrial protein translation would communicate to the cells to prepare for an increase in cellular metabolism. Coordination with the nucleus would be required to handle this increased metabolic load, such as the upregulation of genes involved in the glycolytic and oxidative–phosphorylation pathways. In conclusion, we hypothesize that the effect of taurine is indirect through the mitochondrial–nuclear genome crosstalk pathway. Taurine may therefore act as a signal to activate metabolic pathways such as glucose catabolism that would increase the substrates to fuel mitochondrial NST.

## 5. Conclusions

In conclusion, we provide evidence to a potential mechanistic role for taurine in the regulation of NST. Following NST activation, taurine levels significantly accumulate in the inguinal adipose depots and its synthesis is driven by all three taurine biosynthetic enzymes of which Ado appears to be the most responsive to NST stimulation. We demonstrate that Ado is critical not only to maintain taurine levels in the cell, but that it is also critical for mitochondrial bioenergetic capacity. Finally, we share our findings that taurine supplementation either directly or indirectly alters the chromatin landscape to enhance a suite of genes that promote functional thermogenesis, which will lead to protection against obesity and metabolic disease. Taurine can be purchased as an over-the-counter supplement and supplementation is not reported to have adverse effects in humans [67,68]. Indeed, taurine is currently being tested in several clinical trials, including trials to continue to define its efficacy in the protection from obesity [69,70,71,72,73]. Defining the molecular function of taurine will be beneficial to leverage this pathway toward the treatment of obesity and metabolic disease.

## Data Availability

Data described in the manuscript will be made available upon request.

## Supplementary Materials

The following supporting information can be downloaded at: https://www.mdpi.com/article/10.3390/nu15163532/s1, Table S1: List of primary antibodies; Table S2: List of primers.
