# Supplementary material for: Ado-Mediated Depletion of Taurine Impairs Mitochondrial Respiratory Capacity and Alters the Chromatin Landscape of Inguinal Adipose Tissue
Source: Nutrients. 2023 Aug 11;15(16):3532. doi: 10.3390/nu15163532 (PMC10458711; doi:10.3390/nu15163532)
Supplement: Supplementary file 1 [file nutrients-15-03532-s001.zip › nutrients-2482741-supplementary.pdf]

Supplementary Materials

Table S1. Antibody.

Antibody Name	Catalog Number
ß-Actin	Cell Signaling Technology #4970
Gapdh	Cell Signaling Technology #8884
Hsc70	Santa Cruz #sc-7298
Cdo	Abcam #ab232699
Csad	Thermo Fisher PA5-97631
Ado	Thermo Fisher PA5-78733
Histone 3	Cell Signaling Technology #12648
Mt-Co1	Abcam #ab14705
Vdac1	Santa Cruz Biotechnology sc-390996
Sdha	Abcam #ab4715
Uqcrfs1	Abcam #ab198392
Ndufa9	Abcam #ab14713
Cox5	Abcam #ab110262
Ucp1	Abcam #ab209483

Table S2. Primers.

Gene Name	Primers
Rpl13 forward primer	5′-*AGAAGGGAGACAGTTCTGCTG*-3′
Rpl13 reverse primer	5′-*ATGCCAAAGAGTCGGGCATT*-3′
Ucp1 forward primer	5′-*ACTGCCACACCTCCAGTCATT*-3′
Ucp1 reverse primer	5′-*CTTTGCCTCACTCAGGATTGG*-3′
Cdo forward primer	5′-*GGTCTCCGGTGACCCTAGT*-3′
Cdo reverse primer	5′-*CATGAATACTGCTGCCGTGC*-3′
Csad forward primer	5′-*CCAGGACGTGTTTGGGATTGT*-3′
Csad reverse primer	5′-*ACCAGTCTTGACACTGTAGTGA*-3′
Ado forward primer	5′-*GGTCACCTACATGCACATCTACG*-3′
Ado reverse primer	5′-*ACAGCACCT TGAGCATACCGT*-3′
Gpi1 forward primer	5′-*CCATCAAGGTGGACGGCAAAGA*-3′
Gpi1 reverse primer	5′-*CCGTGATGGATTTGCCAGTGTAC*-3′
Mito DNA forward primer	5′-*CTAGAAACCCCGAAACCAAA*-3
Mito DNA reverse primer	5′-*CCAGCTATCACCAAGCTCGT*-3
B2M forward primer	5′-*ATGGGAAGCCGAACATACTG*-3
B2M reverse primer	5′-*CAGTCTCAGTGGGGGTGAAT*-3
